# New Insights into the Role of Oxidative Stress Mechanisms in the Pathophysiology and Treatment of Multiple Sclerosis

**DOI:** 10.1155/2016/1973834

**Published:** 2016-10-18

**Authors:** Bożena Adamczyk, Monika Adamczyk-Sowa

**Affiliations:** Department of Neurology in Zabrze, Medical University of Silesia, ul. 3 Maja 13-15, 41-800 Zabrze, Poland

## Abstract

Multiple sclerosis (MS) is a multifactorial disease of the central nervous system (CNS) characterized by an inflammatory process and demyelination. The etiology of the disease is still not fully understood. Therefore, finding new etiological factors is of such crucial importance. It is suspected that the development of MS may be affected by oxidative stress (OS). In the acute phase OS initiates inflammatory processes and in the chronic phase it sustains neurodegeneration. Redox processes in MS are associated with mitochondrial dysfunction, dysregulation of axonal bioenergetics, iron accumulation in the brain, impaired oxidant/antioxidant balance, and OS memory. The present paper is a review of the current literature about the role of OS in MS and it focuses on all major aspects. The article explains the mechanisms of OS, reports unique biomarkers with regard to their clinical significance, and presents a poorly understood relationship between OS and neurodegeneration. It also provides novel methods of treatment, including the use of antioxidants and the role of antioxidants in neuroprotection. Furthermore, adding new drugs in the treatment of relapse may be useful. The article considers the significance of OS in the current treatment of MS patients.

## 1. Introduction

Multiple sclerosis (MS) is a multifactorial disease of the central nervous system (CNS) in which both inflammatory and neurodegenerative processes occur simultaneously. In the course of the disease inflammation is decreased whereas the degeneration of the CNS progresses [1]. Several forms of MS are distinguished. These are the following: RRMS (relapsing-remitting MS), SPMS (secondary progressive MS), and PPMS (primary progressive MS). In RRMS an inflammatory process predominates whereas in SPMS and PPMS a neurodegenerative process is more strongly expressed. Relapses defined as episodic exacerbations of neurological signs or symptoms are characteristic of RRMS [2]. Between the relapses patients fully or partially recover from the deficit. The most common form of MS is RRMS which may progress into SPMS after years of remission; SPMS is a natural consequence of RRMS in which relapses decrease and finally disappear over time. In PPMS relapses do not occur from the onset of MS. Progressive axonal loss typically accompanies the PPMS form [3]. As a result, in the course of progressive forms the dominance of neurodegeneration is related to a lack of functional improvement [4, 5].

The ultimate causative factor of this process remains unknown. However, it is suspected that the development of MS may be affected by genetic and environmental factors. Recent observations confirm the fact that oxidative stress (OS) became an important factor associated with the development of demyelination [1, 6, 7].

## 2. The Importance of OS in MS

The inflammatory component in MS is important not only due to axonal and neuronal loss but also due to the fact that it starts the degenerative cascade in the early stage of MS [6]. Interestingly, persistent hyperactivation of oxidative enzymes suggests an “OS memory” in chronic neuroinflammation [62]. The induction of the activation of microglia and mitochondrial dysfunction plays a particular role in inflammatory processes. Microglia activated by T-lymphocytes release proteolytic enzymes, cytokines, oxidative products, and free radicals. On the other hand, microglia have a number of protective properties [59] such as a positive role in the promotion of neuroprotection, lowering inflammation, and stimulation of tissue repair [60].

The development of neurodegeneration in MS is a complex process that may be related to primary apoptosis, synaptopathy, and excitotoxicity associated with glutamate overload, ionic channel dysfunction, calcium overload, mitochondriopathy, proteolytic enzyme production, and activation of apoptotic pathways. It is also important that mitochondrial dysfunction results in an increased production of reactive oxygen species (ROS), which is detrimental to neurons and glia [12, 61]. On the other hand, OS damages the mitochondria, which disrupts the transport of adenosine triphosphate along the axon, and consequently leads to neurodegeneration [63–65]. Importantly, the neurodegenerative process is complicated and still poorly understood.

For a better understanding of the redox processes in the course of MS, some general issues related to OS need to be addressed.

### 2.1. The Mechanisms of OS

The brain tissue is very sensitive to the action of radicals due to its high demand for oxygen and a limited possibility of obtaining antioxidants. An imbalance between free radical production and antioxidative defense results in OS and nitrosative stress [18, 66].

A free radical can be defined as an unstable, short-lived, and highly reactive atom or molecule [18]. Free radicals, ROS and reactive nitrogen species (RNS), can affect important classes of biological molecules, thus leading to multiple lipid and protein damage via peroxidation and nitration processes [18].

**Figure 1:**
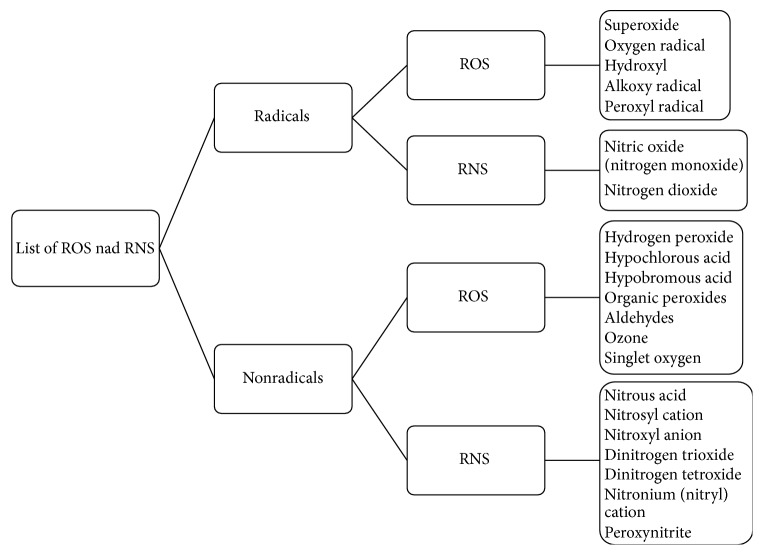
Reactive oxygen species (ROS) and reactive nitrogen species (RNS) [5, 59–61]. The classification of ROS and RNS depended on having an unpaired electron. Nonradial species exists without an unpaired electron.

The levels of ROS/RNS are involved in various physiological functions such as the immune function (i.e., defense against pathogenic microorganisms). They are also involved in a number of cellular signaling pathways, in the mitogenic response, and in the redox regulation [18, 67, 68]. Both ROS and RNS can be classified into two groups, that is, radicals and nonradicals [8, 10, 18, 69] (Figure 1).

The endogenous sources of ROS include mitochondria, peroxisomes, endoplasmic reticulum, and phagocytic cells. Macrophages constitute a major factor responsible for the production of ROS [10] due to high oxygen consumption [6, 18].

The redox reaction involves mainly a superoxide radical, hydrogen peroxide, hydroxyl radical anion, nitric oxide (NO), peroxynitrite [10, 13], peroxyl radical, singlet oxygen, ozone, and hypochlorous acid [18]. Some of these free radicals are described in more detail below.

The superoxide radical exists in two forms such as superoxide or hydroperoxyl radical anion [18]. The enzymes that can produce superoxide include xanthine oxidase [63], lipoxygenase, cyclooxygenase [9, 21], and nicotinamide adenine dinucleotide phosphate- (NADPH-) dependent oxidase [30].

Hydrogen peroxide is formed by the enzyme superoxide dismutase (SOD). It can easily penetrate the biological membranes and damage DNA by producing the hydroxyl radical [29]. The hydroxyl radical can strongly react with both organic and inorganic molecules [17].

Nitric oxide is generated by different nitric oxide synthases (NOSs). Three types of NOS isoforms are distinguished, that is, neuronal NOS (nNOS), endothelial NOS (eNOS), and inducible NOS (iNOS). Nitric oxide is an important intracellular second messenger [18]. It is involved in a number of biological functions such as blood pressure regulation, smooth muscle relaxation, neurotransmission, defense mechanisms, and immune regulation [11].

Peroxynitrite, which is highly toxic, is formed by the reaction between the superoxide radical and NO (nitrogen monoxide) [61]. During the subsequent reaction, new reactive compounds lead to oxidation processes of lipids, proteins (methionine and tyrosine), and DNA [24].

### 2.2. Redox Processes in MS

Researchers suggest that dysregulation of axonal bioenergetics plays a critical role in OS and subsequent axonal injury [25, 64].

Interestingly, the examination of the cerebrospinal fluid (CSF) during exacerbation of MS revealed a bioenergetic failure that was associated with an increased mitochondrial proton leak and an increased expression of genes involved in oxidative damage [35–37]. Moreover, the presence of proinflammatory cytokines in the CSF and prooxidative markers (e.g., nitrotyrosine) led to cytokine-induced synaptic hyperexcitability and consequent glutamate-dependent neurotoxicity [34, 40].

Recent studies suggest the importance of ceramides in the CSF as signaling molecules leading to impaired mitochondrial function. The short-chain ceramides stimulated oxygen species production and led to neuronal death [23, 39].

Iron accumulation in the brain is of great importance. This process leads to chronic cell stress, resulting in axonal and neuronal death [70]. Abnormal iron accumulation was found in MS plaques. Extracellular hemoglobin oxidized and led to local OS through the globin radical which was responsible for myelin basic protein oxidative cross-linking and heme involved in lipid peroxidation [70].

The process of neurodegeneration depends on the liberation of iron from the myelin sheath during demyelination [22]. A diffuse neurodegenerative process is related to the high iron content in the basal ganglia [20]. Ferrous iron may strengthen oxidative injury in the presence of oxygen radicals produced by the oxidative burst [16, 26, 33]. Oxidative stress, mitochondrial injury, and energy failure may be involved in plaque formation and neurodegeneration in white and grey matter lesions [41, 56, 71]. Another scientific report suggested that neurodegeneration in MS was associated with chronic subclinical extravasation of hemoglobin into lesions, the dysfunction of different cellular protective mechanisms against extracellular hemoglobin reactivity and OS [57].

Other studies stressed that alterations in the oxidant/antioxidant balance contributed to the pathophysiology of MS. Consideration was given to the balance between the concentration of compounds such as lipid peroxidation levels, carbonyl protein content, DNA damage and SOD, CAT activities, vitamins C and E, and nonprotein thiol content [42]. Also, the presence of free radicals in the nervous tissue may be toxic; for example, peroxynitrite increases inflammation and in the chronic phase leads to such a high concentration that it may lead to neurodegeneration [13].

Due to a constant lack of useful markers of the disease, it is important to find compounds whose levels are easy to mark, which may bring vast clinical implications. The review of the literature presented below is an attempt to collect biomarkers.

### 2.3. Markers of OS for Assessment: Serum, Erythrocytes, CSF, Saliva, Urine

Free radicals can damage biological molecules including nucleic acids, proteins, and lipids [18, 66]. The products of these reactions can become markers of OS. Serum is the most common material for the evaluation of the components of OS. It allows the estimation of most enzymes, substrates, and products of redox reactions. These enzymes include xanthine oxidase, NOS, lipoxygenase, cyclooxygenase, myeloperoxidase [47], prolyl oligopeptidase [51], nicotinamide adenine dinucleotide phosphate-oxidase 1 (NOX1), and NADPH-dependent oxidase [30]. The following may become the markers of oxidative lipid damage: isoprostanes (IsoP-prostaglandin like substances), for example, 8-iso-prostaglandin (F2*α*-8-iso-PGF2*α*) which constitutes the product of lipid peroxidation of arachidonic acid, malondialdehyde (MDA) [18], the formation of fluorescent peroxidized lipid-protein covalent adducts, and the increase in conjugated diene [70]. Oxidative stress involves the oxidation of proteins and glycoxidation. The following are the results of this reaction: the glycophore content, the total level of advanced protein oxidation (AOPP), protein carbonyls, dityrosine level, N′-formylkynurenine, and a decreased level of serum protein thiol groups [55].

The other specific markers of protein oxidation such as tyrosine (a marker for hydroxyl radical) and 3-nitrotyrosine (a marker for RNS) [43] are also considered. Furthermore, 3-nitrotyrosine is a specific marker of peroxynitrite-induced cellular damage [18].

Another study showed a possibility of using parameters such as ketodienes and Schiff bases [46]. Other indicators in the serum included kynurenine, N′-formylkynurenine, thioredoxin [32], and 8-hydroxy-2′-deoxyguanosine [47].

The parameters used for the measurement of the overall level of OS among healthy individuals and patients with MS are as follows: the total oxidant status (TOS), the oxidative stress index (OSI), and the total antioxidant status (TAS). This reflects the overall level of OS. In turn, OSI is defined as the ratio of TOS to TAS. The total antioxidant status shows the overall level of antioxidant capacity of the human body [72]. The oxidative stress index determines the oxidant/antioxidant balance more reliably in the body.

Following further research, more mediators may be determined in the serum, such as thiobarbituric acid reactive substances, advanced oxidation protein products, fructosamine [73], and activated *α*-2-macroglobulin level [51]. Both AOPP and MDA may be also marked in erythrocytes [74, 75]. Furthermore, the following are also measured in the CSF: levels of MDA and IsoP [54, 76, 77], ceramides [78], chemokine 11 (CCL11) [79], AOPP, and a decreased level of total thiol groups [80].

Both saliva and urine may serve as a useful material. The levels of thiobarbituric acid reactive substances and advanced glycation end-products can be assessed in saliva [73]. Urine may be a good material for the assessment of urinary 8-iso-PGF2*α*, which is a marker of lipid peroxidation in vivo [81].

Researchers reported that acrolein (2-propenal) might strengthen OS [59]. Acrolein antibody strategy was indicated to measure local acrolein levels but quantification of urinary 3-hydroxypropylmercapturic acid (3-HPMA) was the best marker for measuring global acrolein accumulation when glutathione levels were not depleted [82].

Other biomarkers are serum levels of IL-6, tumor necrosis factor (TNF-*α*), interferon *γ* (IFN-*γ*), IL-4, IL-10, and IL-17, albumin, ferritin, plasma levels of lipid hydroperoxides (CL-LOOH), carbonyl protein, and nitric oxide metabolites (NOx) [83]. Lipophilic fluorescent end-CL-products of free radicals may be an interesting marker [84]. Oxidative stress is also evaluated by tert-butyl hydroperoxide-initiated chemiluminescence [85] or the formation of ROS from 2′,7′-dichlorodihydrofluorescein-diacetate fluorescence [86].

The oxidative stress factors possible for assessment in serum, erythrocytes, CSF, saliva, and urine are collectively presented in Table 1.

### 2.4. Linking OS Markers with Disease Course, Relapses, Disability, and MRI Lesions

A number of researchers try to find OS markers which are connected with MS. The review of the literature shows that there are some OS markers of the disease course; for example, studies demonstrated a significant increase in the levels of 8-iso-PGF2*α* in the CSF of patients with SPMS [86]. Guan et al. showed high levels of urinary 8-iso-PGF2*α* (a marker of lipid peroxidation in vivo) in MS patients. The concentration of urinary 8-iso-PGF2*α* was significantly higher in patients with SPMS and PPMS as compared to the control group [81]. It was also shown that a reduced level of prolyl oligopeptidase activity and activated *α*-2-macroglobulin level in the plasma were characteristic of PPMS and RRMS [51]. In addition, in another study plasmatic AOPPs were equally higher in RRMS and SPMS patients [87].

Recently acrolein, an endogenously produced toxic compound and a pathological factor in MS, underwent analysis [88]. It may be used as a potential biomarker for diagnosis and prognosis of MS [82].

Results from the study of Fiorini et al. showed that proteins such as hemopexin, alpha-1-B glycoprotein, inter-a-trypsin inhibitor heavy chain H4, complement C3, and antithrombin III were found to be more oxidized in pathological samples. Oxidation of these proteins might be used to determine the level of OS in the body [1].

Recent scientific findings, which offer the first evidence of increased RNA oxidation in normal-appearing cortex of MS brain, seem to be of great importance. However, further studies are needed to clarify the role of RNA oxidation in MS brain [89].

Cerebrospinal fluid IsoP, which is an OS marker of relapse, was higher in patients with a first clinical attack suggestive of MS as compared to the controls [77]. In turn, lower vitamin D levels may be responsible for the development of MS [42]. It may be related to vitamin D-binding protein (DBP) which was found to be more oxidized in both remitting and relapsing phases. However, higher levels of oxidation rate of DBP were observed during relapses. The increased oxidation rate of DBP already observed in the remitting course showed that some molecular pathways were not completely suppressed during remission as compared to the controls [1].

The degree of oxidation of clusterin was upregulated in MS patients as compared to the healthy controls [1]. This protein was responsible for chaperone-activity already increased in the remitting phase and kept upregulated in the relapsing phase [1].

Karlík et al. reported higher levels of thiobarbituric acid reactive substances and advanced glycation end-products in the saliva of patients with relapse. The study also demonstrated an increase in other OS markers of relapses in the plasma such as thiobarbituric acid reactive substances, advanced oxidation protein products, and fructosamine [73].

Oxidative stress markers of disability included platelet hemostatic function which was advanced in SPMS patients and positively correlated with an increased production of the superoxide radical and with the Expanded Disability Status Scale (EDSS). It seems that the platelet function is activated by a high level of OS [90].

Furthermore, in one of the studies it was demonstrated that inflammation, oxidative and nitrosative stress biomarkers such as serum levels of IL-6, TNF-*α*, IFN-*γ*, IL-4, IL-10, and IL-17, albumin, ferritin, and plasma levels of CL-LOOH, carbonyl protein, AOPPs, NOx, TRAP, and NcoI TNF*β* genotypes might be considered potential predictive biomarkers of high disability in MS (EDSS) and were associated with different aspects of disease progression (higher pyramidal symptoms, sensitive symptoms, and cerebellar symptoms) [83].

Another study, including 110 patients with MS, demonstrated that patients with insulin resistance (IR) had a higher level of disability (EDSS), higher levels of interleukin IL-6 and IL-17 and OS evaluated by tert-butyl hydroperoxide-initiated chemiluminescence and AOPPs compared to patients without IR. It appears that IR and adiposity could contribute to more OS and disability [85].

Other studies indicated that the EDSS and gadolinium enhancement lesion volume-Gd+ were affected by increased levels of OS in erythrocytes in CIS, RRMS, and SPMS patients (increased level of AOPP and MDA) [74, 75].

Summarizing the above observations, the following may become OS markers of disability (EDSS): advanced platelet hemostatic function and increased level of AOOPs and MDA in erythrocytes and plasma levels of CL-LOOH, carbonyl protein, AOPPs, NOx, and TRAP. Additionally, IL-6, IL-17, TNF-*α*, IFN-*γ*, IL-4, IL-10, IL-17, and OS were evaluated by tert-butyl hydroperoxide-initiated chemiluminescence.

Data on OS and MRI lesions reported that a higher level of AOPP and a decreased level of total thiol groups in the plasma and in the CSF were involved in the clinically isolated syndrome (CIS) and RRMS pathophysiology but not with total T2 weighted lesions number and Gd enhancement lesion volume [80].

## 3. The Importance of Antioxidants in MS

Oxidative stress at each stage of MS is a key element in the pathogenesis of the disease. At the time of relapse all these processes are intensified, leading to the loss of neurons over years. Current treatment is focused on decreasing inflammation, however only partially on preventing neurodegeneration. It is possible that a new target of treatment will focus on neutralizing free radicals. The course of the disease is affected by the use of antioxidants and substances that affect antioxidant pathways which reduce the severity and cause faster remission and less pronounced course of neuroinflammation and neurodegeneration [76, 91].

The following is a short description of antioxidants and their practical application in MS.

### 3.1. Enzymatic and Nonenzymatic Antioxidants

Antioxidants, which are divided into enzymatic and nonenzymatic, are substances that protect the body against free radicals (Table 2). Among enzymes the most important include catalase (CAT), glutathione peroxidase (GPx), glutathione reductase (GR), superoxide dismutase (SOD), serum paraoxonase, arylesterase [45], and *δ*-aminolevulinate dehydratase (*δ*-ALA-D) activity [42]. Superoxide dismutase has three isoforms that is, copper/zinc SOD (SOD-1), manganese SOD (SOD-2), and extracellular EC-SOD [13]. It needs to be stressed that in the serum the major antioxidant enzymes include CAT, GPx, peroxiredoxins [18, 92, 93], glutathione-S-transferases (GSTs), and nitrite reductase NAD(P)H quinone oxidoreductase 1 (NQO1) [38]. The concentration of these enzymes in the serum may reflect the status of an antioxidant line of defense.

Nonenzymatic antioxidants may be classified into low molecular weight and antioxidant elements (ions). Low molecular weight antioxidants include uric acid (UA), vitamins C, D, and E, glutathione, coenzyme Q, and b-carotene [13]. Iron (Fe), copper (Cu), zinc (Zn), and manganese (Mn) are the most important ions with antioxidant properties. The general and nonprotein thiol groups represent a nonenzymatic segment of the antioxidant defense system [46].

### 3.2. Antioxidant Factors Possible for Assessment: Serum, Erythrocytes, CSF, Saliva, and Urine

The following can be assessed in the serum: UA [13], nonprotein thiol groups [42, 46], and the total glutathione and reduced glutathione [47]. The lowest molecular weight antioxidants can be used. Other markers of antioxidant ability in the body may be determined in the CSF, for example, concentration of Klotho (an antiaging protein) [94] and total thiol groups [80]. Uric acid may be determined in the CSF; however its concentration depends on the leakage of UA molecules from the serum through the blood-brain-barrier (BBB) and the balance between consumption and production within the CNS [27].

Furthermore, an impaired iron metabolism plays a major role in the pathogenesis of MS [18]. One of a few studies reported that in the saliva of patients with MS ferric reducing ability (FRA) was decreased by 38% as compared to the controls [73]. Ferroxidase (FeOx) activity of ceruloplasmin prevents OS by promoting the connection of free radicals from iron ions to transferrin. A reduced serum FeOx activity was noted in 69 RRMS patients and in 62 patients with other inflammatory neurological disorders [95]. To summarize, FRA can be measured in saliva [73] whereas the ferroxidase (FeOx) activity may be determined in the serum [95].

Erythrocyte SOD and GPx can be marked in erythrocytes [74]. Urine assessment is a noninvasive method useful in the measurement of the oxidative status. Gholipour et al. showed that urine 6-sulphatoxymelatonin (aMT6s, major metabolite of melatonin) levels were significantly lower in MS patients as compared to the control group [96].

Two parameters appear to be significant, that is, the total radical-trapping antioxidant parameter (TRAP) and the total antioxidant status (TAS). The first parameter may be measured by a fluorescence-based method (TRAPm) or calculated (TRAPc) by a mathematical formula, considering antioxidant levels in the serum, that is, protein-bound thiol groups, UA, and vitamins E and C [97]. What is important, TRAP was recently proposed to measure the antioxidant level in the plasma [83]. The difference between TRAPm and TRAPc is due to antioxidants, which are still unidentified [97]. Furthermore, TAS can be determined in the serum and it reflects the overall level of antioxidant capacity of the patient [45].

The antioxidant factors possible for assessment in serum, erythrocytes, CSF, saliva, and urine are collectively presented in Table 3.

### 3.3. The Impact of External and Internal Antioxidant Factors on the Course of Disease, Disability, and MRI Lesions

It was reported that melatonin (10 mg daily/30 days) caused a statistically significant increase in antioxidative enzymes such as SOD and GPx and a decrease in MDA in erythrocytes of SPMS patients [74]. This suggests the possibility of a positive impact of melatonin on the course of severe forms of MS. Glutathione is an antioxidant in the brain which might be a marker of the oxidative line of defense in MS patients and also might serve to monitor the disease course [31].

Another study showed that an expression of antioxidant power such as plasmatic ferric reducing ability (FRA) and thiol group dosage were significantly lower in patients with active disease [87], which may worsen the prognosis. Interestingly, the GSTP1 polymorphism and quinone oxidoreductase 1 (NQO1) variant genotypes in MS patients suggested that a defective function of detoxification enzymes might be a determinant of susceptibility and the clinical manifestation of the disease [38].

One of the studies examined coenzyme Q10 and antioxidized low-density lipoproteins (anti-oxLDL) antibodies, which may help to maintain the BBB integrity and might result in a mild disease course [98].

Aleagha et al. indicated that a decreased concentration of Klotho in the CSF of patients with RRMS showed a significant negative correlation with the disability [94]. Recent reports indicated that decreased urine aMT6s levels significantly correlated with the MS Functional Composite Score. The authors believe that there might be some new hope in developing a quantitative and objective measure to assess the severity of MS [96]. The urine aMT6s levels were not correlated with the level of disability measured by the EDSS scale [96].

Serum UA concentrations in 30 MS patients and 20 controls with noninflammatory neurological diseases supported the significance of UA in the pathogenesis of MS. Serum UA concentrations were found to be significantly lower in MS patients as compared to the controls [27]. However, CSF UA concentrations might not be a reliable marker of disease activity in MS which was assessed by MRI lesions and the CSF/serum albumin quotient [27].

On the other hand, the relationship between disability, MRI Gd + lesions and SOD concentration in erythrocytes in CIS and RRMS patients is not clear and requires further studies [74, 75].

### 3.4. Opportunities for Antioxidant Supplementation in MS: What Can Be Supplemented?

Neuroprotection seems to be a vast area to explore, common to a number of neurodegenerative diseases, including MS. The role of OS in MS appears to be vital. Currently, a large therapeutic potential lies in antioxidants. Research focuses on finding new substances with antioxidant properties.

The melatonin supplementation appears to be useful [74, 75, 99] and may scavenge the hydroxyl, carbonate, alkoxyl, peroxyl, and aryl cation radicals and stimulate the activities of antioxidative enzymes (GPx, SOD, etc.) [74]. It was confirmed that melatonin also plays an important role in improving the antioxidant defense in MS through upregulation of sirtuin 1 (SIRT1) and its target genes for MnSOD and CAT [100]. Moreover, melatonin is selectively taken up by mitochondrial membranes, which makes it a potential therapeutic tool in treating neurodegenerative disorders [50].

In vitro studies demonstrated that dihydroasparagusic acid prevented lipopolysaccharide-induced production of neurotoxic mediators such as NO, TNF-*α*, prostaglandin E2, inducible NOS, cyclooxygenase-2 protein expression, and lipoxygenase activity in microglial cells [101].

Oxidative stress is also responsible for depletion of n-3 polyunsaturated fatty acid (PUFA), leading to disruptions in the lipid-based signaling, intracellular signal dysfunction and increased neurotoxicity. Consequently, n-3 PUFA supplementation is a rational therapeutic approach [102].

*α*- (alpha-) Lipoic acid (ALA) is a natural, endogenous antioxidant that acts as a peroxisome-proliferator-activated receptor-*γ* (PPAR-*γ*) [49]. The researchers showed that increased *δ*-ALA-D activity may be a protective agent against OS [42]. The data provided the first evidence that ALA might increase the production of PPAR-*γ* in vivo in experimental autoimmune encephalomyelitis (EAE) and might reveal antioxidative and immunomodulatory mechanisms for the application of ALA in human MS [49]. Khalili et al. suggested that 1200 mg of lipoic acid daily improved serum TAC among RRMS patients without affecting other biomarkers [103]. On the other hand, one of the systematic reviews showed that over-the-counter antioxidants such as epigallocatechin-3-gallate and ALA offered benefits, however only in preclinical studies. There is no evidence that they alter MS relapses or the disease progression [14].

Interestingly, a randomized study conducted on a sample of 24 patients with RRMS proved that supplementation of coenzyme Q10 for 12 weeks resulted in an increase SOD activity, plasma TAC, and a decrease in MDA levels as compared to the controls [104].

Idebenone, an organic compound known as a synthetic analog of coenzyme Q10, was proven to be beneficial in Friedreich's ataxia and Leber's hereditary optic neuropathy. In these diseases idebenone protected neuronal HT22 cells from glutamate-induced death in vitro. Fiebiger et al. reported that the histopathological examination of the CNS of idebenone-treated mice showed no improvement in inflammation, demyelination, or axonal damage. It seems that this is not a preferred method of MS treatment [105].

It was also shown that serum lipophilic antioxidants, that is, *γ*-tocopherol, *β*-carotene, and coenzyme Q10 were deficient or moved within the border of lower physiological value in a vast majority of MS patients. Researchers suggested that the deficit of lipophilic antioxidants in the blood of MS patients could have a negative impact on bioenergetics of reparative demyelinating processes and could promote neurodegeneration [25].

### 3.5. Antioxidants of Plant Origin

Ghaffari et al. reported that treatment with two doses of saffron extract (5 and 10 *μ*g/rat) weekly resulted in a growth of total antioxidant reactivity capacity, lipid peroxidation products, and antioxidant enzymes activity in the hippocampus of experimental models of MS compared to the controls [106]. Other studies demonstrated that the administration of Nigella sativa seeds in EAE induced in Wistar rats suppressed inflammation, enhanced remyelination in the cerebellum, and reduced the expression of transforming growth factor beta 1 (TGF *β*1) [107]. In turn, a nanodroplet formulation of pomegranate seed oil (denominated nano-PSO) dramatically reduced oxidation of lipids in the brains of rats [48, 108].

A small number of MS patients (*n* = 9) demonstrated a protective effect of hypericum perforatum, which resulted in an increase in neutrophil GPx activity and a decrease in intracellular free calcium ions [109]. Some studies indicated that a low fat diet and antioxidant supplements could reduce levels of free radicals [110–112]. Dietary flavonoids have a potential to protect neurons against OS, an ability to suppress neuroinflammation and modulate cell signaling pathways [112]. Flavonoids such as luteolin, quercetin, and fisetin at concentrations of 20–80 *μ*M decrease the amount of myelin phagocytosed by macrophages [113].

Other studies described a number of potential antioxidants, such as cerium oxide nanoparticles, and sulforaphane, which is an organosulfur compound present in vegetables, ginseng, hemp seed, and evening primrose oils [114–116]. Matrine (MAT) is another antioxidant of plant origin. It is a quinolizidine alkaloid derived from the herb Radix Sophorae Flave. In EAE MAT treatment significantly upregulated the expression of the transcription factor such as nuclear factor (erythroid-derived 2) like 2 (Nrf2) which plays a role in inhibiting OS [58].

Following further studies, synthetic inhibitors of phospholipase A2 (PLA2) from plants including curcumin, ginkgo biloba, and* Centella asiatica* extracts were also used for the treatment of neurological disorders [108].

## 4. Inflammatory Mediators and Antioxidants

New findings suggest that chemokine 11 (CCL11) in the serum and in the CSF released from activated astrocytes promoted OS via microglial NOX1 activation and glutamate-mediated neurotoxicity. These findings proposed using inhibitor of NOX1 in therapy [79]. Another study explained how TNF-*α* inhibited the differentiation of progenitor cells. The effect depended on a number of factors such as increased ROS production, altered mitochondrial calcium uptake, mitochondrial membrane potential, and respiratory complex I activity. The accumulation of progenitor cells at the lesion sites was observed in MS patients [117] and suggested that failed remyelination was a consequence of the inhibition of differentiation [118–120]. In another study, authors presented the possibility of using a TNFR2 agonist as a factor protecting oligodendrocyte progenitor against OS [121].

Scientists suggested that enhanced astrocytic peroxisome proliferator-activated receptor gamma coactivator 1-alpha (PGC-1*α*) levels reduced the production of proinflammatory mediators such as IL-6 and chemokine (C-C motif) ligand 2 and increased the expression of antioxidant enzymes, including peroxiredoxin-3 and thioredoxin-2 in generated human primary astrocytes. Activation of PGC-1*α* may be a protective factor for neurons [44].

The results from the study of EL Andaloussi et al. presented the use of exosomes, biologically active nanovesicles (30–120 nm) that could be easily delivered across the BBB [122] as an improvement to induce postinjury remyelination processes. They stimulated primary dendritic cells cultures with low-level IFN*γ*. Exosomes (IFN*γ*-DC-Exos) contain microRNA species which are involved in oligodendrocyte development pathways and can increase baseline myelination, reduce OS, and improve remyelination. Researchers also found that IFN*γ*-DC-Exos increased oxidative tolerance and antioxidant levels in microglia and potentially included anti-inflammatory miRNAs. Furthermore, IFN*γ*-DC-Exos nasally administered to animals increased CNS myelination in vivo [123].

## 5. The New Possibilities in the Treatment of MS: Neuroprotection

A number of substances are tested for a possible ability to protect the brain against neurodegeneration. In addition, the development of neuroprotective drugs is more problematic [6]. A limited response to the application of ROS scavengers results from their short half-life, on the order of milliseconds and the degree of instability of ROS [3, 59, 124, 125].

Hydralazine may become a potential target for therapy due to the fact that it protects cells from the damaging effects of acrolein [59, 126–128].

Novel agents/approaches could offer help in preventing mitochondrial dysfunction and in improving neurodegeneration. The following are considered: CDDO-ethyl amide, CDDO-trifluroethylamide, pioglitazone, rosiglitazone, resveratrol, 5-aminoimidazole-4-carboxamide ribonucleotide (AICAR), and bezafibrate [129].

Other findings suggested that neural stem cells (NSCs) exposed to 125 *μ*M H_2_O_2_ for 30 min and pretreated with different doses of lovastatin for 48 h were protected against OS-induced cell death by the expression of PGC-1*α*, which is a master regulator of mitochondrial function controlling energy metabolism and Nrf2. It is possible that in the future lovastatin may be used to promote the survival rate of NSCs [130]. The former group which can readily cross the BBB includes simvastatin, atorvastatin, and cerivastatin while hydrophilic statins include rosuvastatin and pravastatin [131].

The experimental results of the effects of exendin-4 and glucagon-like peptide-1 (GLP-1) in several mouse models of MS were reported by Hölscher. The main inflammatory responses were much reduced, as well as the intensity of demyelination. The cytokine release in the spleen was also reduced. It was shown that most GLP-1 mimetics such as exendin-4, liraglutide, and lixisenatide crossed the BBB and showed neuroprotective effects [132, 133]. However, further studies are needed to clarify the relationship with OS.

Novel treatments can be rated in EAE, a mouse model of MS. One of the scientific reports showed the effect of polymerized form of nanocurcumin (PAP) on EAE, which might have a therapeutic effect as an anti-inflammatory and antioxidative stress agent with significant effects on myelin repair mechanisms [133]. Using nontoxic inhibition of myeloperoxidase might restore the BBB integrity thereby limiting migration of myeloid cells into the CNS that drive EAE pathogenesis. These inhibitors may be an effective therapeutic agent for the treatment of MS [134]. Yun et al. discovered a new molecule with a neuroprotective activity, that is, antioxidant protein peroxiredoxin 6 (PRDX6), which can reduce the inflammation in the CNS and potentiate oligodendrocyte survival [135]. The modulation of glutamate release and transport may also become a new therapeutic target [136].

The process, known as “remote damage” may have a significant effect on neurodegeneration. This process can damage neurons functionally related to the primary focus for months and years after the original damage such as stroke, multiple sclerosis, amyotrophic lateral sclerosis, and traumatic injury to the brain and the spinal cord. “Remote damage” may be defined as a variety of pathological processes, such as apoptosis, inflammation, glial activation, oxidative damage, neuronal changes in receptor mosaics, and autophagy. The impact of these factors is important at different times. Viscomi et al. attempted to investigate this process using the hemicerebellectomy (HCB) experimental paradigm. The researchers presented the idea of new therapies based on blocking “remote damage.” The therapeutic window that occurs between the primary and secondary damage can be used to implement new neuroprotective treatment [137–139].

As it can be seen, not all the studies on the role of antioxidants in MS are consistent and further research should be done to test new substances for their effectiveness.

## 6. The Relationship between Immunomodulatory Therapy, OS, and Antioxidants

Immunomodulatory therapies are used to protect from relapses whereas corticosteroids are commonly used in the acute treatment of relapses.

The relationship between OS and dimethyl fumarate (DMF) is partially explained. The transcription factor (Nrf2) is a key regulator of antioxidative defense. Oral DMF activates anti-inflammatory and antioxidative pathways to upregulate the expression of this molecule [15, 52, 140]. A differential expression is involved in the defense against OS, predominantly in actively demyelinating white matter lesions [140–142]. Treatment of oligodendrocytes with DMF induces changes in citric acid cycle intermediates, glutathione, and lipids, indicating that this compound can protect oligodendrocyte metabolism and provide protection from OS [140].

Dimethyl fumarate and monomethyl fumarate (MMF), the immediate metabolite of DMF, activate Nrf2 transcriptional pathways. Target genes of Nrf2 include glutamate cysteine ligase transcription factor 1 and NAD(P)H oxidoreductase-1, resulting in cytoprotective effects against oxidative cellular injury. It is a potential novel mode of action differentiating this drug from other immune-modifying drugs [143]. This mechanism explains the possibility of using this drug in other degenerative diseases of the CNS, such as Parkinson's disease [144]. It is possible that dimethyl fumarate activates the prostaglandin EP2 receptor and consequently inhibits the progression of MS via the cAMP signaling pathway. This is another recently discovered mechanism of action which needs to be clarified due to OS in MS [145]. Dimethyl fumarate attenuates overproduction of ROS, which results in a decrease in a lipid peroxidation product, 7-ketocholesterol induced by ROS. It can protect murine oligodendrocytes 158N (myelin synthesizing cells) against apoptosis and autophagy. It may be responsible for neuroprotection [53]. Another effect of DMF may be associated with the activation of heme oxygenase-1 [146]. The use of a signaling pathway of the Kelch-like ECH-associated protein 1-nuclear factor erythroid 2-related factor 2-antioxidant-responsive element (Keap1-Nrf2-ARE) in vivo and in vitro leads to the downregulation of OS and inflammation, activated by DMF which may protect the nervous tissue against subarachnoid hemorrhage induced brain injury in rats [147]. In addition, it is suspected that DMF could strengthen the BBB by targeting interendothelial junctions in an Nrf2-dependent manner, thus protecting against cerebral edema during ischemic stroke [148]. On the other hand, in one of the studies on the Nrf2 pathway DMF enhanced the severity of lung carcinogenesis in mice [149].

It was also shown that therapies aiming at stimulating endogenous antioxidant pathway, for example, by inducing the Nrf2 pathway [65], may be quite effective in a situation of moderate OS such as the one in classical EAE models. However, they might be ineffective or even counterproductive in the case of extensive oxidative injury. It was proposed that the amplification of oxidative injury in MS was only reflected to a limited degree in the studied rodent models [150].

A new study reported that treatment with fingolimod reduced hyperoxia-induced OS, activation of microglia, and associated proinflammatory cytokine expression in neonatal oxygen-induced brain injury. The thesis could in part explain the efficacy of fingolimod in MS, in which OS plays an essential role [151].

IFN*γ* can damage myelin by spreading depression as in migraine with aura. In contrast, the physiological level of IFN*γ* as produced by environmental enrichment protected against demyelination and OS and was associated with a moderate and phasic increase in a number of proinflammatory cytokines. The controlled administration of pulsed IFN*γ* to brain slice cultures imitating environmental enrichment reduced OS, increased the concentration of myelin basic protein, and reduced spreading depression. Furthermore, stimulation of brain slice cultures with IFN*γ* induced the release of exosomes that had most likely neuroprotective functions [152].

Some studies attempted to prove the efficacy of IFN-*β* which was connected with other immunomodulatory therapies or antioxidant therapies. For instance, it was reported that treatment with IFN-*β* and glatiramer acetate significantly reduced TNF-*α*. However, it did not affect other ROS/NRS biomarkers or disease progression [83].

In another study the level of protein carbonyls was elevated in RRMS patients treated with interferon *β*-1b and glatiramer acetate, whereas the serum protein thiol groups were decreased. Following the same study in RRMS patients without immunomodulatory therapy the same markers of OS were significantly elevated [55]. Sadowska-Bartosz et al. demonstrated an increase in oxidation parameters in the serum in RRMS patients treated with interferon *β*-1a and interferon *β*-1b. However, this increase was less significant compared to untreated RRMS patients or SPMS patients treated with mitoxantrone [32].

Treatment with the combination of glatiramer acetate and N-acetylcysteine had a favorable safety profile. Moreover, it had a positive effect on the redox state [153]. The administration of glatiramer acetate to female BALB/c mice under stress conditions resulted in normalization of ROS levels, restored nNOS activity, and resulted in clinical improvement in learning [154].

Another study demonstrated that melatonin supplementation at a dose of 5 mg over 90 days resulted in a significantly decreased MDA concentration in INF-*β* and glatiramer acetate-treated groups, however not in the mitoxantrone-treated group. In turn, a significant increase in SOD activity was observed only in glatiramer acetate-treated group as compared to the controls [155]. Interestingly, melatonin may also have implications for the treatment of severe MS. One of the studies indicated that the TAC level was significantly lower in the mitoxantrone-treated group and it increased after melatonin supplementation [156].

Using C-phycocyanin, a biliprotein from Spirulina platensis with antioxidant, anti-inflammatory, and cytoprotective properties and INF-*β* improved the redox status in mice with EAE, although they also differentially modulated another subset of genes. C-phycocyanin mainly modulated the expression of genes related to remyelination, gliogenesis, and axon-glia processes, which may be significant in neuroregeneration [157]. Therefore, a combined use of immunomodulatory therapies with antioxidants may prove beneficial.

Attempts were also made to explain some of the beneficial effects of natalizumab and its antioxidant capacity. Researchers studied serum melatonin levels in 18 patients with RRMS treated with natalizumab and noted that it caused significant increases in serum melatonin concentrations [158]. In one of the studies 22 MS patients were assigned to the treatment with 300 mg of natalizumab. After 14 months it was observed that natalizumab prompted a decrease in oxidative-damage biomarker levels, induced nuclear translocation of Nrf2 which was responsible for the activation of the antioxidant pathway, and a fall in serum vascular cell adhesion molecule-1 levels [47]. In addition, it was found that a decrease in carbonylated protein levels was connected with the patients with the highest levels of severity in the process (EDSS > 5) and treatment with natalizumab [159]. Consequently, it resulted in an increase of antioxidants and a reduction in OS biomarkers.

It should be borne in mind that mitoxantrone is potentially associated with an increased level of OS but on the other hand, the study demonstrated that mitoxantrone did not have an effect on the activity of paraoxonase 1 (an enzyme that protects the cell from OS) [160].

Arnold et al. evaluated the suicidal erythrocyte death induced by mitoxantrone. The study proved that mitoxantrone triggered cell apoptosis, partially due to the formation of ROS and ceramide thus increasing OS. Additionally, the authors assessed the effect of adding the antioxidant N-acetylcysteine, which significantly reduced the effect of mitoxantrone [86].

Due to the fact that the studies were not conclusive, it appears that treatment with interferon and mitoxantrone does not reduce OS [32].

To conclude, it appears that most of the drugs used in MS are directly or indirectly associated with OS.

## 7. Corticosteroids in Relapses: The Importance of OS and Antioxidants

The role of corticosteroids in OS is poorly understood. Wang et al. examined levels of MDA and TAC in peripheral blood and the CSF of RRMS patients in 7 days before methylprednisolone (MP) treatment and one month after MP treatment. They found that the increase in OS markers preceded inflammatory response in MS patients and MP treatment reduced the neuroinflammatory attack by decreasing brain antioxidant enzymes [54].

Ozone autohemotherapy is an emerging therapeutic technique that can change the brain metabolism. It was recently shown that MS patients demonstrated a marked increase in cytochrome-c-oxidase (CYT-c) activity and concentration about 40 minutes after the end of the autohemotherapy, possibly revealing a reduction of the chronic OS level typical of MS sufferers [161].

A protective effect of ozone (O_3_) therapy was reported in EAE in rats either alone or in combination with corticosteroids. Such a combination allowed reducing the dose of MP due to a decreased level of brain glutathione, paraoxonase 1 enzyme activity, brain MDA, TNF-*α*, IL-1*β*, IFN-*γ*, Cox-2 immunoreactivity, and p53 proteins [162]. The study showed that adding compounds that modulate redox pathways in the cell could increase the effectiveness of the therapy and reduce a dose of corticosteroids.

## 8. Conclusions

The brain tissue, with a considerable number of phospholipid membranes, is very sensitive to the action of radicals due to a significant presence of mitochondria and consequently massive oxygen metabolic processes. A number of studies document the participation of OS in MS pathophysiology. Oxidative stress processes participate in both inflammatory and neurodegenerative pathophysiological components of MS. Oxidative stress is associated with the dysregulation of axonal bioenergetics, cytokine-induced synaptic hyperexcitability, abnormal iron accumulation, and the oxidant/antioxidant balance. Markers of OS assessed in the serum, erythrocytes, CSF, saliva, and urine may have diagnostic properties whereas antioxidants may have clinical application in the future. There are at least a couple of OS markers of the disease course which can be particularly useful in the diagnosis of severe forms of MS such as SPMS and PPMS. Other useful applications include markers of relapse and OS markers of disability. There might be some new hope in an objective assessment of the severity of MS.

Many antioxidants may have a positive impact on the course of MS. These substances include melatonin, dihydroasparagusic acid, n-3 polyunsaturated fatty acid (PUFA), *α*- (alpha-) lipoic acid (ALA) and others (including plant origin antioxidants). Innovative therapies are aimed in particular at neuroprotection and neurodegeneration. Potential drugs include compounds such as hydralazine, exendin-4, glucagon-like peptide-1 (GLP-1), and also lovastatin which protects against OS-induced cell death by the expression of PGC-1*α* and Nrf2.

Currently, a number of new studies focus on the immunotherapy and OS. Natalizumab and fingolimod have a positive effect on antioxidant capacity and may result in a reduction in OS markers. The relationship between DMF and OS is best known and is associated with the modulation of OS molecules. On the other hand, mitoxantrone is a drug that may be responsible for an increase in OS. There are new suggestions combining mitoxantrone therapy with antioxidant supplementation such as N-acetylcysteine and melatonin in order to alleviate the toxicity of mitoxantrone.

Summarizing, using OS markers as biomarkers of MS severity or relapse could be a long-awaited helpful diagnostic tool. Moreover, adding antioxidants to immunotherapy which is well-established in MS may be reasonable and highly beneficial for MS patients due to their ability to reduce OS. Further research should be done to test new antioxidants for their effectiveness.

## Figures and Tables

**(a):** 

The biological material: serum											
Enzyme	Reference	Lipid damage	Reference	Protein damage	Reference	RNA/DNA damage	Reference	Carbohydrate damage	Reference	Other	Reference
Xanthine oxidase	[8]	Isoprostanes	[5]	Nitrotyrosine	[5, 9]	8-Hydroxy-2′-deoxy guanosine	[10]	Fructosamine	[11]	Peroxynitrite	[12]
NADPH dependent oxidase	[13]	MDA	[5, 14, 15]	O-Tyrosine	[9]					TOS	[16, 17]
Monoamino oxidase	[18]	Fluorescent peroxidized lipid-protein covalent adducts	[19, 20]	Glycophore	[21]					OSI	[17]
Alpha-ketoglutarat dehydrogenase	[18]	CL-LOOH	[22]	AOPP	[21–27]					Thiobarbituric acid reacting substances	[11]
Glycerol phosphate dehydrogenase	[18]	Conjugated diene	[19]	Protein carbonyls	[21, 22]					*α*-2-Macroglobulin	[13]
Lipoxygenase	[10]			Dityrosine	[21]					Acrolein	[28]
Cyclooxygenase	[10]			N′-Formylkynurenine	[21, 29]					Schiff bases	[30]
Myeloperoxidase	[10]			Kynurenine	[29]					Lipophilic fluorescent end-CL-products	[20]
Prolyl oligopeptidase	[31]			Decreased proteins thiol groups	[21, 32]					Tert-Butyl hydroperoxide-initiated chemiluminescence	[26]
Nitric oxide synthase	[31]									2′,7′-Dichlorodi: hydrofluorescein: diacetate fluorescence	[33]
										NOx	[22]
										CCL11	[34]
										IL-6	[22, 26]
										IL-17	[22, 26]
										TNF-*α*	[22]
										IFN-*γ*	[22]
										IL-4	[22]
										IL-10	[22]

**(b):** 

The biological material								
	CSF	References	Erythrocyte	References	Urine	References	Saliva	References
Lipid damage	Isoprostanes 8-iso-PGF2*α* MDA	[35–37] [38] [35–37]	MDA	[24, 25]	8-Iso-PGF2*α*	[39]		

Protein damage	AOPP Total thiol groups	[21, 23] [21, 23]	AOPP	[24, 25]			Advanced glycation end-products	[11]

Other	Ceramides CCL11	[40] [34]			3-Hydroxy propyl-mercapturic acid (acrolein metabolic)	[28]	Thiobarbituric acid reacting substances	[11]

SOD: superoxide dismutase, NOX1: nicotinamide adenine dinucleotide phosphate-oxidase 1, MDA: malondialdehyde, CL-LOOH: lipid hydroperoxides, NOx: nitric oxide metabolites, TOS: total oxidant status, OSI: oxidative stress index, TNF-*α*: tumor necrosis factor, IFN-*γ*: interferon *γ*, AOPP: advanced oxidation protein products, 8-iso-PGF2*α*: isoprostane, CCL11: chemokine 11, and CSF: cerebrospinal fluid.

**Table 2:** The types of antioxidants. The types of antioxidants depend on molecular structure. The table lists the most important barrier antioxidant enzymes and other compounds and ions which are not enzymes.

Enzymes oxidants [16, 29, 41–44]	Nonenzymatic antioxidants [13]	
	Low molecular weight antioxidants	Antioxidant elements
CAT GPx GR SOD Paraoxonase Arylesterase GSTs NQO1 Peroxiredoxin-3 Thioredoxin-2, 6 FeOx *δ*-ALA-D	Uric acid Vitamin C Vitamin D Vitamin E Glutathione Coenzyme Q B-Carotene AU	Ions: Cu, Fe, Zn, Mn

CAT: catalase, GPx: glutathione peroxidase, GR: glutathione reductase, SOD: superoxide dismutase, GSTs: glutathione-S-transferases, NQO1: NAD(P)H:quinone oxidoreductase 1, FeOx: ferroxidase, *δ*-ALA-D: *δ* aminolevulinate dehydratase, and AU: uric acid.

**Table 3:** The Biomarkers of antioxidant capacity. The serum is assayed for enzymes with antioxidant properties. There are also other compounds and important parameters which can be assessed in the serum. In addition, new possibilities for the use of other biological materials occurred. All these markers provide knowledge about the antioxidant status of the organism.

The biological material											
Serum	References		References	Saliva	References	CSF	References	Erythrocytes	References	Urine	References
*Enzymes*		*Other*									
CAT	[31, 45]	TAC	[22, 46]	FRA	[11, 16]	Klotho protein	[47]	SOD	[24, 25]	aMT6s	[46]
GPx	[8, 48]	TRAP	[11, 14, 16, 47, 49, 50]			Total thiol groups	[21, 23]	GPx	[24]		
GR	[8]	FRA	[27]			AU	[51]				
SOD	[13–15]	Nonproteins thiol group	[30, 47]								
Paraoxonase 1	[45, 160, 162]	*γ*-Tocopherol	[54]								
Arylesterase	[16]	*β*-Carotene	[12, 54]								
GSTs	[42]	Coenzyme Q10	[54]								
NQO1	[42]	Ceruloplasmin	[55]								
Peroxiredoxin-3	[5, 56–58]	Ferritin	[5]								
Thioredoxin-2, 6	[29]	AU	[12]								
FeOx	[55]										
*δ*-ALA-D	[41]										

GPx: glutathione peroxide, SOD: superoxide dismutase, CAT: catalase, GPx: glutathione peroxidase, GR: glutathione reductase, SOD: superoxide dismutase, GSTs: glutathione-S-transferases, NQO1: NAD(P)H:quinone oxidoreductase 1, FeOx: ferroxidase, *δ*-ALA-D-*δ*: aminolevulinate dehydratase, TAC: total antioxidant capacity, TRAP: total radical-trapping antioxidant parameter, FRA: ferric reducing ability, AU: uric acid, aMT6s: 6-sulphatoxymelatonin levels, CSF: cerebrospinal fluid.
